# Left Atrial Diverticula Present in the Right Lower Pulmonary Vein Thrombus Attachment Area

**DOI:** 10.7759/cureus.53422

**Published:** 2024-02-01

**Authors:** Hidekazu Takeuchi

**Affiliations:** 1 Internal Medicine (Cardiology), Takeuchi Naika Clinic, Ogachi-Gun, JPN

**Keywords:** left atrium diverticulum, tee, cardiac ct, right lower pulmonary vein thrombi, pulmonary vein thrombosis

## Abstract

Left atrial diverticula (LADs) are thought to be associated with atrial fibrillation and an ischemic brain state. However, the mechanisms of LAD formation are unknown. Pulmonary vein thrombi (PVTs) can cause acute myocardial infarction (AMI) and ischemic stroke by releasing rather large particles. Additionally, PVTs can release much smaller particles, including neutrophil extracellular traps (NETs) and/or other components of NETs, such as DNA and histones. To treat these diseases, it may be crucial to know the specific traits of PVTs. However, these issues are not direct effects of PVTs on the left atrium (LA). It is unclear whether PVTs affect the LA directly. We checked the direct effects of PVTs on the LA using cardiac computed tomography (CT) and transesophageal echocardiography (TEE). The patient was a 73-year-old female with hypertension. TEE revealed extended LA thrombi from the right lower pulmonary vein, which were attached to the anterosuperior wall of the LA. Cardiac CT revealed the attaching area as a defect of enhancement and dimly revealed LAD with full thrombi on the attaching area. It was difficult to recognize the LAD at first; however, after one month of standard-dose heparin-warfarin treatment, the LAD was clearly detected using cardiac CT. LA thrombi could not be detected using cardiac CT.

## Introduction

Left atrial diverticula (LADs) are not rare, and their incidence has been reported to be approximately 10~40% [1-5]. Relationships between LAD and atrial fibrillation and ischemic brain alterations have been reported [1]. LADs often appear in the anterior and superior walls of the left atrium (LA) [1-3,5]. Compared with females, males are more likely to have LADs [5]. Atrial fibrillation has a high incidence in LAD patients [4,5]. However, the mechanisms of LAD formation are unknown. Pulmonary vein thrombosis is common in elderly patients with chest pain [6] and underestimated. Each patient has several thrombi in pulmonary veins [7,8], indicating pulmonary vein thrombosis is common in elderly patients with age-related disease. When a patient has a respiratory infection, neutrophil extracellular traps (NETs) are generated, which are thought to produce arterial thrombi (ATs) to interfere with the spread of the pathogen through the pulmonary vein [7]. ATs eventually become larger and become pulmonary vein thrombi (PVTs). We reported that smaller PVTs become larger PVTs [8], which subsequently extend into the LA [9] and finally attach to the LA wall [10,11].

PVTs can cause acute myocardial infarction (AMI) and ischemic stroke by releasing rather large particles; PVTs release much smaller particles, including NETs and other components of NETs, such as DNA and histones. NETs are associated with many diseases, such as type 2 diabetes mellitus (T2DM) [12], atherosclerosis [13,14], decreased blood flow [15], heart failure [16], and cancer [17,18]. PVTs may be associated with these diseases by releasing NETs, DNA, and histones. These issues are not direct effects of PVTs on the LA. The direct effect of PVTs on the LA is not known.

We checked the direct effect of PVTs on the LA using cardiac computed tomography (CT) and transesophageal echocardiography (TEE).

## Case presentation

A 73-year-old female with hypertension was assessed for PVT. She had no symptoms of chest pain, fever, cough, sputum, or cerebral infarction. The cardiac exam did not reveal arrhythmia or heart murmur. The respiratory exam did not reveal decreased breath sounds, lung crackles, or wheezing. The height was 161 cm and the weight was 49 kg. A chest roentgenogram revealed no lung cancer or cardiomegaly. No previous treatment with warfarin had been performed. ECG indicated sinus rhythm, complete right bundle branch block, and a heart rate of 91 beats/minute. The serum D-dimer level was 0.7 μg/ml (normal: < 1.0 μg/ml), the activity of protein S was 99.4% (normal: 74-132%), and the activity of protein C was 111% (normal: 64-135%). The homocysteine level was 11 nmol/mL (normal: 5-15 nmol/mL). The C-reactive protein (CRP) level was 0.06 mg/dL (normal: 0.00-0.19 mg/dL). To scrutinize the thrombi in the LA and pulmonary veins, cardiac CT and TEE were performed.

**Figure 1:**
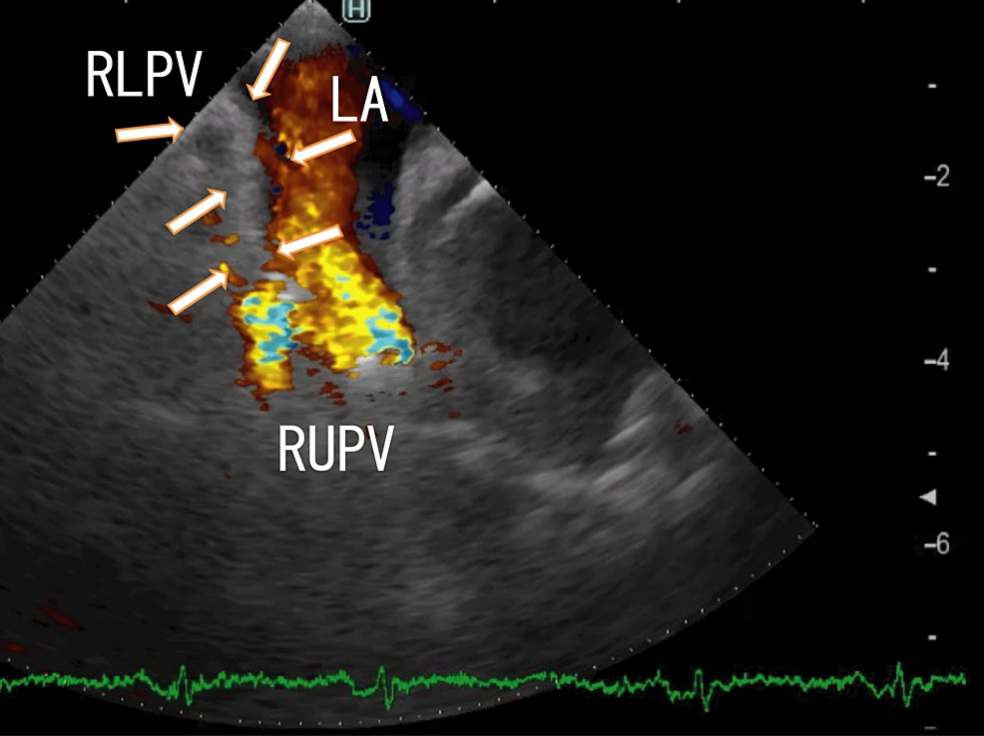
TEE images demonstrating that LA thrombi extended from RLPV thrombi TEE images demonstrating thrombi in the LA as thick line-like shapes connected to the entrance of the RUPV (arrows). The thrombi were not white and were thick and linear in shape. The blood flows from the RUPV are shown as mixtures of blue, yellow, and dark red areas. LA, left atrium; RLPV, right lower pulmonary vein; RUPV, right upper pulmonary vein

**Figure 2:**
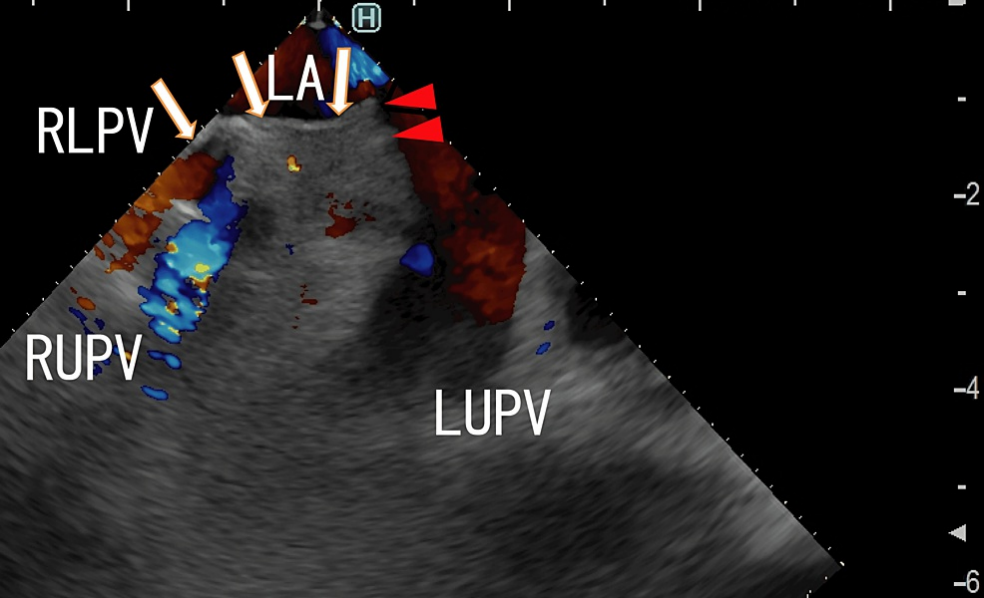
TEE images demonstrating thrombi in the left atrium TEE images demonstrating thrombi in the LA as white line-like shapes that appeared to be connected to the entrance of the RUPV (arrows). The thrombi included a large white area and were linear in shape. The left end of the thrombi was attached to the wall of the LA (arrowheads). The blood flows from the RUPV and LUPV are shown as red areas. A portion of the blood flow from the RUPV is shown as blue areas. LA, left atrium; LUPV, left upper pulmonary vein; RLPV, right lower pulmonary vein; RUPV, right upper pulmonary vein

TEE demonstrated thrombi in the LA as line-like shapes that extended from the right lower pulmonary vein (RLPV) thrombi. The left end of the thrombi was attached to the anterosuperior wall of the LA (Figure 1, 2).

**Figure 3:**
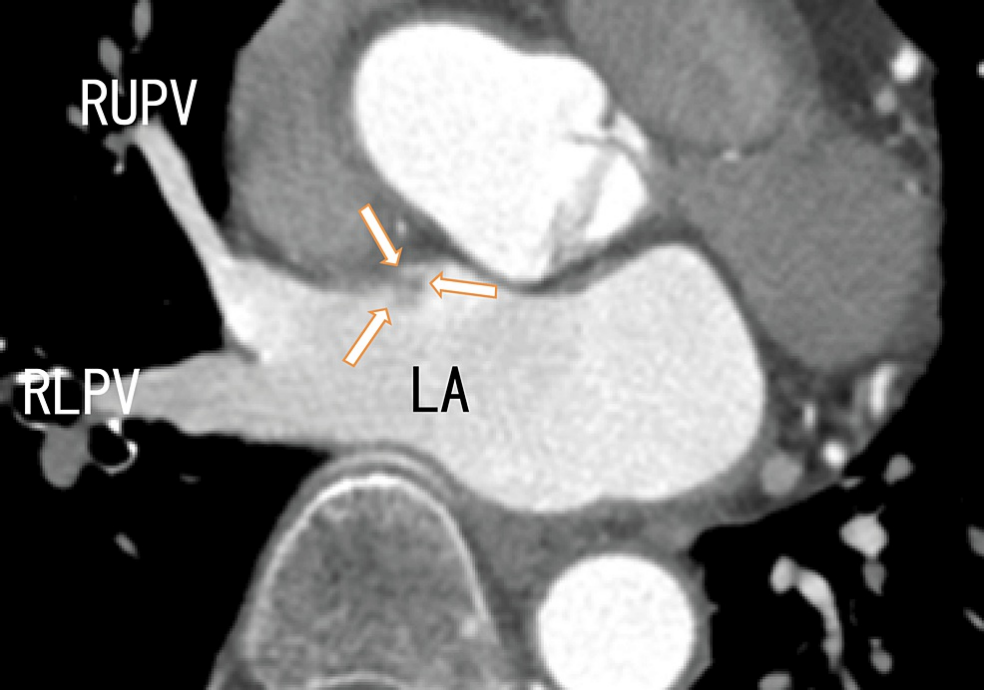
Axial images from an 80-MDCT scan revealing the RUPV, RLPV, and LA There were no images of thrombi in the LA. There was a rather dark area in the RLPV, and there was a dark line in the center of the RUPV, indicating that there were thrombi in both the RUPV and the RLPV. Additionally, there was a strongly dark area in the LA (arrows), suggesting that both extended RLPV thrombi and the anterior wall of the LA were attached. LA, left atrium; RLPV, right lower pulmonary vein; RUPV, right upper pulmonary vein, MDCT, multidetector computed tomography

Cardiac CT demonstrated no thrombi in the LA; however, there were dark areas on the anterosuperior wall of the LA, which should be attached areas. The RLPV appeared darker, indicating thrombi in the RLPV. A dark line was in the center of the RUPV, suggesting the presence of pulmonary vein thrombi (Figure 3).

**Figure 4:**
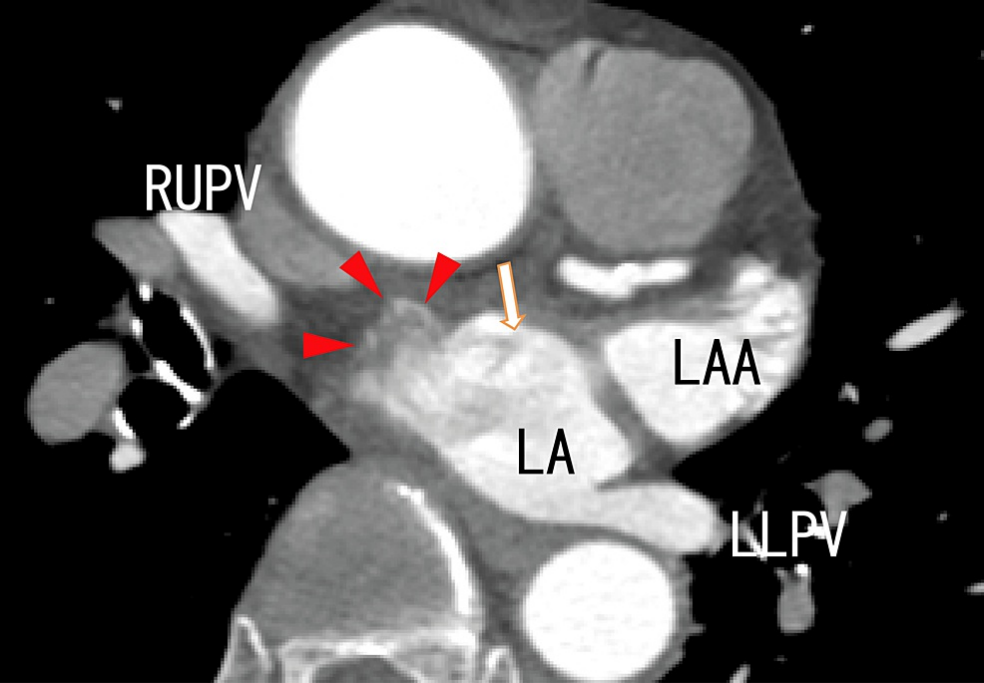
Axial images from an 80-MDCT scan revealing the RUPV, LLPV, and LA There was a rather dark area (arrow), and there were some images of thrombi in the LA as the dark area. There were some enhanced areas on the anterior wall of the LA wall (arrowheads), suggesting that the diverticulum was full of thrombi. LA, left atrium; LAA, left atrial appendage; LLPV, left lower pulmonary vein; RUPV, right upper pulmonary vein; MDCT: multidetector computed tomography

The superior area of the LA had a wide variety of darker areas, indicating that there were many thrombi. On the superior side of the LA, there were some strange dark and white areas. However, they could be diverticula of the LA, which was full of thrombi (Figure 4).

**Figure 5:**
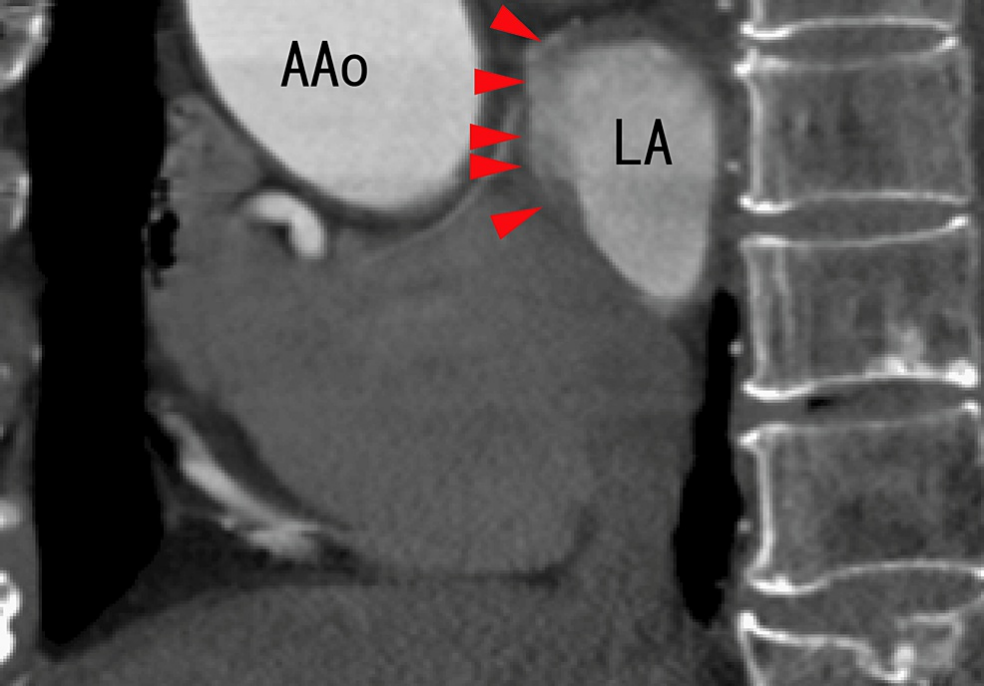
Sagittal images from an 80-MDCT scan revealing the LA and LAD There were more dark areas than LAs (arrowheads) on the anterior wall of the LA, suggesting that the diverticula were full of thrombi. The images of thrombi on the upper side of the LA are shown as darker areas. AAo, ascending aorta; LA, left atrium; MDCT, multidetector computed tomography

On the anterior side of the LA, there were some strange dark and white areas. The lesions could be diverticula of the LA, which was full of thrombi (Figure 5).

**Figure 6:**
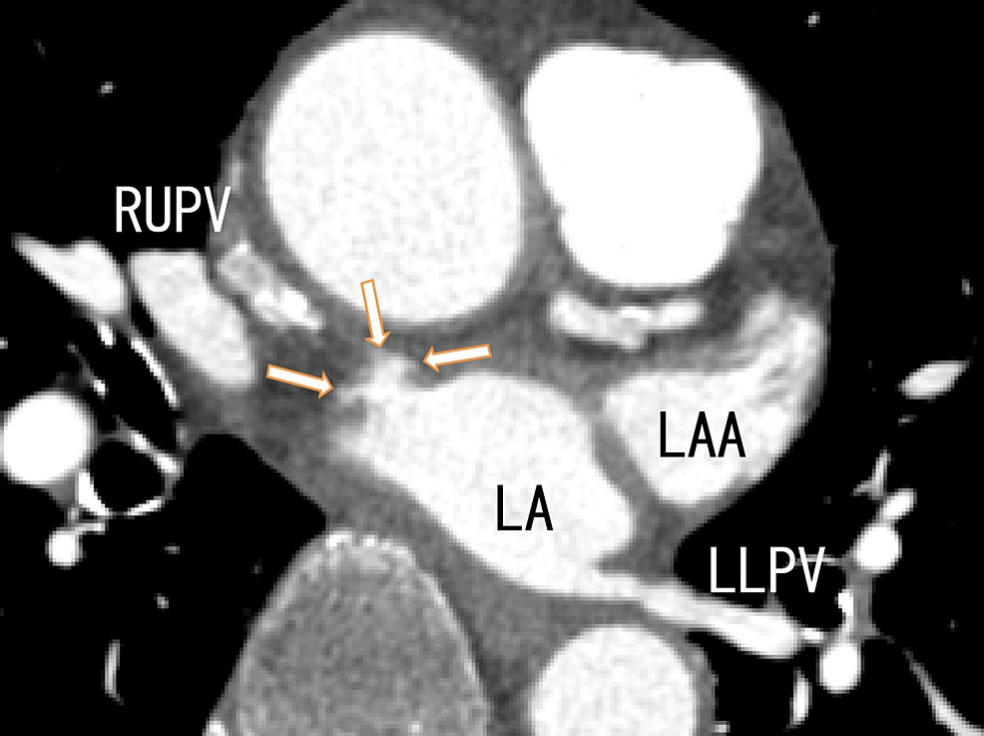
Axial images from an 80-MDCT scan obtained after one month of heparin and warfarin treatment The location was similar to that of the 80-MDCT scan images in Figure 4. There was a diverticulum on the anterior wall of the LA (arrows). There was a little dark area in the LA, which was situated at the root of the diverticulum. The dark areas of the RUPV and LA became clearer, indicating that the thrombi had resolved. LA, left atrium; LAA, left atrial appendage; LLPV, left lower pulmonary vein; RUPV, right upper pulmonary vein; MDCT, multidetector computed tomography

**Figure 7:**
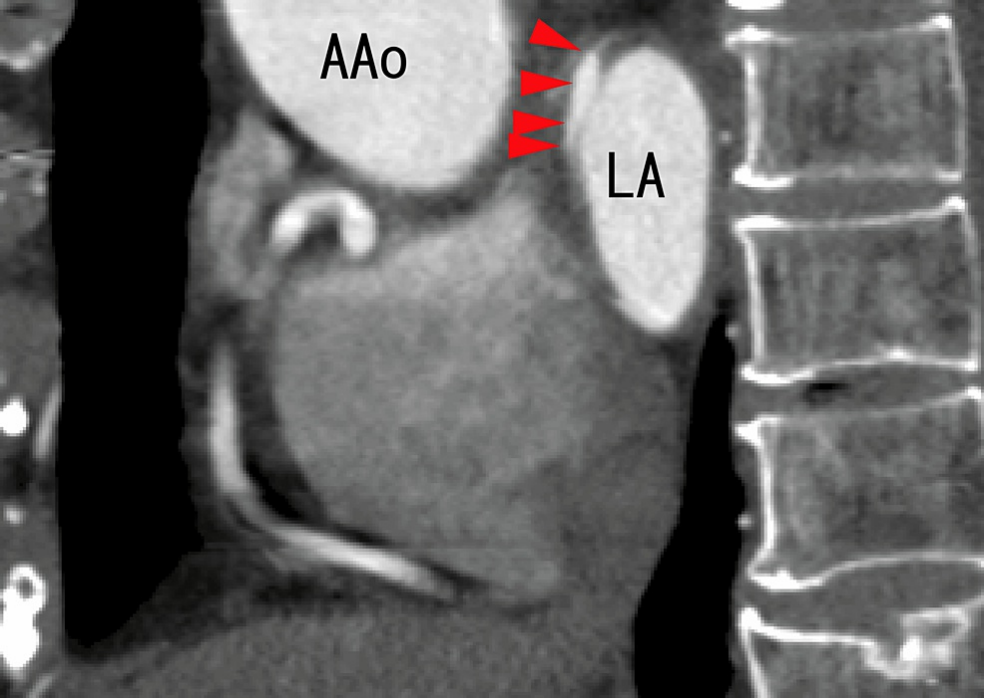
Sagittal images from an 80-MDCT scan obtained after one month of heparin and warfarin treatment The location was similar to that of the 80-MDCT scan images in Figure 5. There was a diverticulum on the anterior wall of the LA (arrowheads). The darker areas of the LA and LAD in Figure 5 became clearer, indicating that the thrombi had resolved. The distance between the anterior wall of the LAD and the anterior wall of the LA became shorter than that in Figure 5, indicating that the LAD decreased. AAo, ascending aorta; LA, left atrium; MDCT, multidetector computed tomography

After one month of treatment with a standard dose of heparin and warfarin, the diverticula clearly appeared in a similar position as some strange structures, as shown in Figure 4 and Figure 5. Most of the LA thrombi in the superior areas of the LA seemed to disappear. The inside of the diverticula seemed to become increasingly clearer (Figures 6, 7).

## Discussion

In this case, thrombi were seen in the LAD that could be resolved by standard-dose heparin-warfarin therapy. After standard-dose heparin-warfarin therapy, the LAD seemed to become rather smaller. The characteristics of the thrombi in the LAD are unknown; however, these thrombi may have a weak structure, and some of the particles in the LAD are easily released, facilitating the diagnosis of AMI or ischemic stroke. However, additional studies are needed to clarify these factors.

Previously, we reported that extended LA thrombi protrude from RLPV thrombi to attach to the LA wall, which was detected using cardiac CT and transthoracic echocardiography (TTE). This is the first case report to use TEE to show that RLPV thrombi extend into the LA and attach to the LA anterosuperior wall. We could depict the attached area in detail using TEE. LADs exist in the attaching areas, indicating that PVTs may be associated with LAD formation.

A retrospective thrombus study revealed that the retrieved thrombi contained white blood cells (WBCs), fibroblasts, and calcifications [19], suggesting that the thrombi were old and indicating that the patients had stable thrombi in the body for a long time. These lesions were likely PVTs. WBCs are associated with inflammation, heart failure, and possibly LA remodeling. However, PVTs might have different types of WBCs that are apt to promote inflammation. Heart failure is associated with inflammation of the heart and heart enlargement. After attaching to the LA wall, PVTs might affect heart function and heart structure. When we find the LAD in cardiac CT images, we need to check for PVTs and LA thrombi using TEE because TTE cannot detect those thrombi properly in many cases, and cardiac CT cannot detect those thrombi in most cases. If we find such thrombi, then we can resolve the thrombi using warfarin, direct oral anticoagulants (DOACs), and heparin [6,9,20]. Such treatment could prevent the occurrence of AMI and ischemic stroke and could prevent LAD formation and LA remodeling. Additionally, such treatment might prevent NET-associated diseases such as T2DM. However, additional studies are needed to clarify these relationships.

## Conclusions

TEE revealed that the RLPV thrombi extended into the LA, after which the thrombi attached to the anterosuperior wall of the LA. Cardiac CT showed that the dim LAD was filled with thrombi positioned on the attachment area. One month of standard-dose heparin-warfarin treatment resolved the LAD thrombi and LA thrombi, and cardiac CT clearly showed the LAD.
